# Resveratrol Induces Apoptosis-Like Death and Prevents *In Vitro* and *In Vivo* Virulence of *Entamoeba histolytica*

**DOI:** 10.1371/journal.pone.0146287

**Published:** 2016-01-05

**Authors:** Jonnatan Pais-Morales, Abigail Betanzos, Guillermina García-Rivera, Bibiana Chávez-Munguía, Mineko Shibayama, Esther Orozco

**Affiliations:** Departamento de Infectómica y Patogénesis Molecular, Centro de Investigación y de Estudios Avanzados del IPN, México DF, México; Stanford University, UNITED STATES

## Abstract

*Entamoeba histolytica* causes amoebiasis, an infection that kills 100,000 individuals each year. Metronidazole and its derivatives are currently used against this protozoan, but these drugs present adverse effects on human health. Here, we investigated the effect of resveratrol (a natural compound) on *E*. *histolytica* trophozoites viability, as well as its influence on the parasite virulence. Trophozoites growth was arrested by 72 μM resveratrol and the IC_50_ was determined as 220 μM at 48 h. Cells appeared smaller, rounded and in clusters, with debris-containing vacuoles and with abnormally condensed chromatin. Resveratrol triggered reactive oxygen species production. It caused lipid peroxidation and produced phosphatidylserine externalization and DNA fragmentation this latter evidenced by TUNEL assays. It also provoked an increase of intracellular Ca^2+^ concentration, activated calpain and decreased superoxide dismutase activity, indicating that an apoptosis-like event occurred; however, autophagy was not detected. Cytopathic activity, phagocytosis, encystment and *in vivo* virulence were diminished dramatically by pre-incubation of trophozoites with resveratrol, evidencing that resveratrol attenuated the trophozoite virulence in vitro. Interestingly, after the inoculation of virulent trophozoites, animals treated with the drug did not develop or developed very small abscesses. Our findings propose that resveratrol could be an alternative to contend amoebiasis.

## Introduction

*Entamoeba histolytica* infects 50 million people and kills 100,000 individuals around the world every year [1]. This protozoan causes diarrhea and hepatic abscesses mainly in poor areas of tropical countries, but *E*. *histolytica* has a worldwide distribution [2,3]. Trophozoites are the invasive form of the parasite and they provoke the symptoms of the disease that could be lethal when it is not properly treated. Cysts are excreted with feces, contaminating water and food that could infect other people [4]. Trophozoites can be *in vitro* cultured, but cysts are not generated consistently in the laboratory, hampering the study of the molecular mechanisms that underly the parasite life cycle, postponing the development of novel strategies to erradicate amoebiasis.

Amoebiasis is mainly treated by metronidazole (1-(ß hydroxyethyl-2-methyl-5-nitroimidazole) and its derivatives. These drugs are toxic to humans, causing nausea, dry mouth, headache and neurotoxicity [5]. In addition, metronidazole-resistant *E*. *histolytica* strains have been generated in the laboratory [6], pointing out the risk of metronidazole-resistant parasites emergence in endemic areas. Besides, metronidazole could be carcinogenic in mice and rats [7]. For these and other reasons, recently, auranofin and extracts from *Codiaeum variegatum* among other compounds have been proposed as alternative drugs against amoebiasis [8,9,10], evidencing that the development of new pharmacological strategies for amoebiasis defeat are still being a priority.

During infection, *E*. *histolytica* trophozoites can migrate from the gut to the liver, lungs, brain, genitals and other tissues in a metastasis model [11]. They present an active membrane exchange during tissues invasion and phagocytosis of target cells. Consequently, trophozoites exhibit high metabolic activity that could make them vulnerable to damage mediated by reactive oxygen species (ROS), as occurs in cancer cells [12].

Resveratrol (trans-3,4',5- trihydroxystilbene) is a phytoalexin, which has been described as harmless to mammals in concentration up of 2 g/Kg [13]. It is widely distributed in foods, such as grapes and nuts [14]. In normal cells, this compound has antioxidant properties and decreases the intracellular ROS production, either by direct scavenging or by activating enzymes that function as antioxidant defenses [15,16]. In contrast, in cancer cells and in some parasites, resveratrol triggers an excess of ROS that may kill them [12,17].

Resveratrol could be an attractive therapeutic option for amoebiasis, conducting trophozoites to ROS production and eventually provoking autophagy or apoptosis [18,19]. Autophagy has not been described in *E*. *histolytica*, but some proteins of the Atg8 conjugation system were already reported in this parasite [20]. On the other hand, apoptosis is characterized by DNA rupture and biochemical imbalance of some metabolic pathways, in which caspase and calpain enzymes play important roles [21]. In contrast to autophagy, apoptosis-like death has been studied in *E*. *histolytica* under stress conditions [22,23]. The parasite has non-canonical caspases [24]; and *E*. *histolytica* genes that could correspond to mammalian and yeast caspase orthologous, are highly divergent [21]. For these reasons apoptosis in *E*. *histolytica* is still recognized as an apoptosis-like phenomenon. However, this parasite possesses calpain-like enzymes [23,24] that could play similar roles to caspases.

We show here that resveratrol triggered apoptosis-like death in *E*. *histolytica* trophozoites, it decreased the *in vitro* virulence of trophozoites and when administered orally, decreased the formation of amoebic liver abscesses in hamsters inoculated with virulent trophozoites.

## Materials and Methods

### *E*. *histolytica* and *E*. *invadens* cultures

Trophozoites of *E*. *histolytica* (HM1:IMSS strain) and *E*. *invadens* were axenically cultured in TYI -S-33 medium at 37°C and 25°C, respectively [25]. Encystment of *E*. *invadens* was performed by incubation of trophozoites in LG47-TYI-S-3 medium [26] and cysts were stained by 0.01% calcofluor [27] and counted under epifluorescence microscope (Nikon Eclipse 80i).

### Evaluation of the resveratrol effect on *E*. *histolytica* trophozoites

Trophozoites were incubated at 37°C with 72,110, 220, 440 and 880 μM of resveratrol in 0.4% ethanol in TYI-S-33 medium and cell number was determined each 12 or 24 h by hemocytometer counting. Diameter of trophozoites was measured directly in pictures. Viability was evaluated by cell motility and shape, as well as by Trypan blue exclusion and by the WST-1 reagent (Roche) to measure cell proliferation [28]. Resveratrol half maximal lethal concentration (IC_50_) was calculated by Reed’s equation [29]. As a positive control, we added 0.5% H_2_O_2_ to the cultures [23] and 0.4% ethanol (the resveratrol vehicle) as internal control. Experiments presented here were performed at least three times in duplicate.

### Flow cytometry experiments

Size and cell granularity of resveratrol-treated trophozoites were determined by flow cytometry (Becton Dickinson FACSCalibur equipment), using a 488 nm Argon laser. Light scattered in the forward direction was roughly proportional to cell size, whereas light scattered at 90° angle was proportional to cell granularity. Data analysis was performed using the FlowJo software.

### Transmission electron microscopy (TEM)

Trophozoites were washed twice with PBS, pH 7.4, fixed with 2.5% (v/v) glutaraldehyde in 0.1 M sodium cacodylate buffer, pH 7.4, for 1 h and post-fixed for 60 min with 1% (w/v) osmium tetroxide. After dehydration with increasing concentrations of ethanol (70, 80, 90 and 100%) and propylene oxide, samples were embedded in Poly/bed^®^ epoxy resin and polymerized at 60°C for 24 h. Thin sections (60–90 nm) were contrasted with uranyl acetate and lead citrate before being examined in a Philips Morgagni 268 D electron microscope (FEI Company).

### Measurement of ROS activity

ROS activity was calculated using 2’,7’–dichlorofluorescein diacetate (DCFDA) reagent (Sigma-Aldrich). Trophozoites (1 x 10^6^) were incubated with 0.4 mM DCFDA for 15 min in the dark, washed with PBS pH 7.4 and analyzed by flow cytometry. Data analysis was performed using the FlowJo software.

### Lipid peroxidation assay

Total lipids were extracted from trophozoites by 1:2 chloroform:methanol (v/v), as described [30]. Lipid peroxidation was determined in lipids using the Peroxidetect^TM^ kit (Sigma-Aldrich). Briefly, 100 μL of lipid extracts were incubated with 1 mL of Working Color Reagent at room temperature (RT) for 30 min and color intensity was measured by spectrophotometry at 560 nm. Standard curves were generated for each experiment using 0 to 16 nmol of *ter*-BuOOH (Sigma-Aldrich). Absolute methanol was used as a blank.

### Annexin V binding experiments

Trophozoites (1 x 10^6^) were washed with PBS, pH 7.4, and re-suspended in 500 μL of binding buffer containing Annexin V-FITC (eBioscience), mixed gently, incubated for 15 min at RT in the dark and washed twice with the binding buffer. Samples were counterstained by propidium iodide (PI) (10 μL/100μL) to analyze membrane permeability and calculate the number of cells with membrane rupture and those with membrane loss of asymmetry, then, they were mounted with Vecta-Shield medium (Vector Laboratories) and observed under a Carl Zeiss LSM 700 laser confocal microscope. In parallel, samples were subjected to flow cytometry. Data analysis was performed by Summit V4.3 software.

### Terminal deoxynucleotidyl transferase dUTP nick end labeling (TUNEL) assays

Trophozoites were fixed with 2% (v/v) formaldehyde for 60 min at RT, washed with PBS, pH 7.4, and permeabilized with 0.1% Triton X-100 and 0.1% sodium citrate, for 2 min on ice, rinsed with PBS, and incubated in 50 μL of TUNEL reaction mixture (In Situ Cell Death Detection kit, fluorescein) (Roche) for 60 min at 37°C in the dark. Trophozoites were washed twice and prepared for examination under laser confocal microscope, as described above. Additionally, samples were subjected to flow cytometry and data analysis was performed by FlowJo software.

### Quantification of cytosolic Ca^2+^

Trophozoites (1 x10^6^/mL) were washed twice with buffer I (116 mM NaCl, 5.4 mM KCl, 0.8 mM MgSO_4_, 5.5 M D-glucose and 50 mM HEPES, pH 7.0), re-suspended in loading buffer (116 mM NaCl, 5.4 mM KCl, 0.8 mM MgSO_4_, 5.5 mM D-glucose, 1.5% sucrose, 50 mM HEPES, pH 7.4), and 6 μM Fluo-4 AM (Molecular Probes), incubated for 1 h at 37°C with occasional agitation and washed with ice-cold buffer I. Then, 250 μL of the trophozoite suspension were diluted into 2.4 mL of buffer I. Fluo-4 AM was measured using 494 and 506 nm emission and excitation filters, respectively, in a Perkin Elmer fluorimeter. [Ca^2+^] was determined using the formula: ${\left[{\text{Ca}}^{2+}\right]}_{\text{i}}=\text{Kd}\left(\frac{\text{F}2-\text{F}3}{\text{F}4-\text{F}1}\right)$, where F1 is the fluorescence signal obtained from the entire cell, F2 represents the fluorescence signal after addition of 1 mM EGTA, F3 is the fluorescence after cell lysis with 0.04% Triton X-100 in 30 mM Trizma base and F4 is the fluorescence after adding 4 mM CaCl_2_. The dissociation constant (Kd) of fluo-4 AM was taken at 345 nM, according to the manufacturer instructions.

### Calpain activity

The polypeptide Suc-Leu-Leu-Val-Tyr-AMC (Sigma-Aldrich) was used to assess calpain activity [31]. Briefly, 40 μL of trophozoites protein extracts (1 mg protein/mL) were added to 160 μL of 50 μM Suc-Leu-Leu-Val-Tyr-AMC in 100 mM Tris/HCl, 145 mM NaCl, pH 7.3. Then, calpain activity was assayed with 10 mM Ca^2+^ 7-amino-4-methylcoumarin (AMC) (Sigma-Aldrich). Released AMC was measured by fluorescence spectroscopy using 360 nm excitation and 430 nm emission filters. Standard curves were generated for each experiment using AMC of known concentrations (0–25 μM). Calpain activity was expressed as pmol of AMC released per mg of total protein at 10 mM Ca^2+^.

### Superoxide dismutase activity (SOD)

SOD activity was determined using SOD Assay Kit (Sigma-Aldrich) according to the manufacturer’s instructions. Briefly, trophozoites protein extracts (1 mg/mL) in 200 μL of working solution and 20 μL of working solution enzyme were incubated 20 min at 37°C. Color intensity was measured by spectrophotometry at 450 nm. Standard curves were generated for each experiment using SOD (Sigma-Aldrich) of known concentrations (0–10 U/mg). Dilution buffer was used as a blank.

### Erythrophagocytosis assays

Freshly obtained human erythrocytes were washed and incubated for 5, 10 and 15 min at 37°C with trophozoites pre-incubated for 12 h in 110 μM resveratrol or with 0.4% ethanol in TY1-S-33 medium (30:1 ratio). Afterwards, trophozoites were incubated with distilled water for 10 min to lyse the adhered, but not ingested erythrocytes. Samples were fixed with 2.5% glutaraldehyde for 30 min at RT. Erythrocytes were stained with diaminobenzidine (8.4 mM) to facilitate counting [32] of ingested erythrocytes. One hundred resveratrol-treated or untreated trophozoites were selected at random to be counted in duplicate experiments that were performed at least three independent times. Means of erythrocytes in 100 trophozoites was obtained for each point and data were analyzed by ANOVA. Before these experiments, trophozoites were examined under the light microscope to verify their shape and movement Viability was also quantified by Trypan blue exclusion and WST-1 assays. Only cultures with viability greater than 95% were used.

### Cytopathic effect of trophozoites on cell culture monolayers

Confluent MDCK cell monolayers were incubated with *E*. *histolytica* trophozoites (20:1 ratio) at 37°C in an environment of 5% CO_2_, during 1 h. Then, epithelial cells were exhaustively washed with cold PBS and the remaining cells in the plates were fixed with 2.5% glutaraldehyde and stained with 1% methylene blue for 10 min. Destruction of MDCK monolayers was measured by color intensity of the remaining cell monolayer, in comparison with control MDCK, that were cells not incubated with trophozoites. Measurements were performed using the ImageJ software.

### Effect of resveratrol on the *in vivo* virulence of *E*. *histolytica* trophozoites

To evaluate the effect of resveratrol on the production of amoebic liver abscesses in animals, we performed three types of experiments, using seven or eight animals per group and a similar number for controls. Syrian golden hamsters (*Mesocricetus auratus*, 4 weeks old, 100 ± 5 g each animal) were anesthetized with 45 mg/Kg of sodium pentobarbital (Anestesal, Pfizer) and intraportally inoculated with 3 x 10^6^ trophozoites with more than 95% viability. For the first assay, trophozoites were pre-treated with 110 μM resveratrol, for 12 h. Untreated or ethanol-treated trophozoites were used as controls. For the second type of experiments, animals were treated with 30 mg of resveratrol (100 mg/Kg), orally administrated each 8 h, 2 days before and 10 days after inoculation of virulent trophozoites. For the third type of experiments animals were inoculated with virulent trophozoites and after 96 h livers were examined to corroborate the presence of amoebic hepatic abscesses in one of the control groups. Other group was treated with 100 mg/Kg resveratrol as described started the treatment protocol after 96 h inoculation of trophozoites. As controls, other animals were treated with water, 50 μl of ethanol or 20 mg/Kg metronidazole each eight hours, following a similar protocol. Ten days after challenge, hamsters were sacrificed by an overdose of sodium pentobarbital according to the guidelines of the 2000 AVMA Panel of Euthanasia. Livers and abscesses were dissected and weighted. Liver damage was calculated as the weight of abscesses formed, divided by the weight of whole liver before the injured areas were removed.

### Tissue collection and processing

Liver samples were fixed in 10% phosphate-buffered formalin and processed for conventional embedding paraffin, then, 4–6 μm thick sections were stained with hematoxylin and eosin [33]. For immunohistochemistry assays, livers were excised, fixed in 4% paraformaldehyde (PFA) and embedded in paraffin. Thin sections (4–6 μm) were obtained deparaffinized and treated with 10% H_2_O_2_ at RT for 60 min to remove endogenous peroxidase. Sections were incubated first with normal goat serum at RT for 60 min and ON at 4°C with a rabbit antibody (1:100) produced by Invitrogen, against a 16 residues polypeptide (431-KYHSNSTYVQFYNHTI-446) obtained from the EhCP112 protease [34]. Then, preparations were incubated with goat anti-rabbit IgG biotinylated-labeled antibody (1:300; Molecular Probes), followed by incubation with streptavidin–biotin–peroxidase complex (Zymed) and diaminobenzidine (DAB) containing 0.04% H_2_O_2._ Antibody biding was revealed by hematoxylin. Rabbit pre-immune serum was used as a negative control. Samples were analyzed using a light microscope (Nikon Eclipse 80i).

### Ethic Statements

The Center for Research and Advanced Studies (CINVESTAV) fulfill the standard of the Mexican Official Norm (NOM-062-ZOO-1999) “Technical Specifications for the Care and Use of Laboratory Animals” based on the Guide for the Care and Use of Laboratory Animals “The Guide”, 2011, NRC, USA with the Federal Register Number BOO.02.03.02.01.908, awarded by the National Health Service, Food Safety and Quality (SENASICA) belong to the Animal Health Office of the Secretary of Agriculture, Livestock, Rural Development, Fisheries and Food (SAGARPA), an organization that verifies the state compliance of such NOM in Mexico. The Institutional Animal Care and Use Committee (IACUC/ethics committee) from CINVESTAV as the regulatory office for the approval of research protocols involving the use of laboratory animals and in fulfillment of the Mexican Official Norm have reviewed and approved the animal care and handle of hamsters used in the virulence assays (Protocol Number 0313–06, CICUAL 001).

### Statistical analysis

The values of all experiments were expressed as mean ± standard error of three independent experiments, each in duplicate. Trophozoites treated with resveratrol or H_2_O_2_ were compared with ethanol-treated or untreated trophozoites using the paired Student’s-test (*p<0.05, **p<0.01, ***p<0.001). In experiments comparing more than two groups we used one-way ANOVA test. Statistical analysis was carried out using the Graphpad Prism V 5.01 software.

## Results

### *E*. *histolytica* trophozoites are sensitive to resveratrol

**Fig 1:**
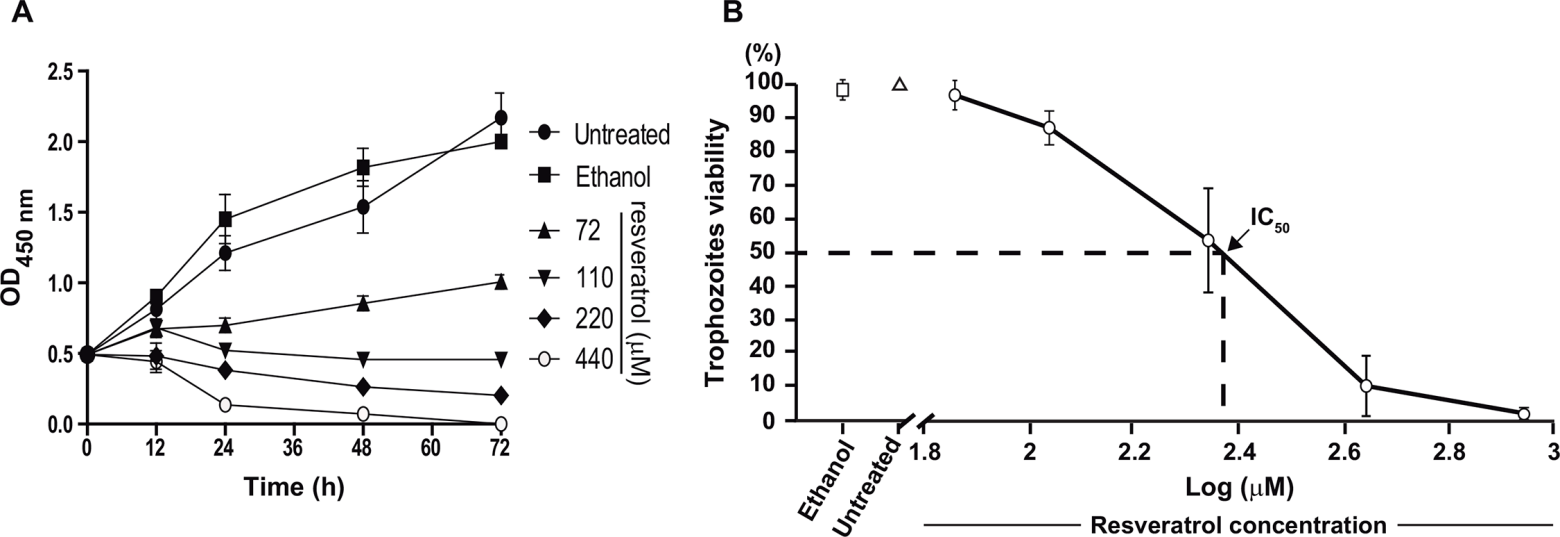
Effect of resveratrol in *E*. *histolytica* trophozoites. **A)** Trophozoites growth was measured at different times by spectrophotometry using the WST-1 reagent. **B)** Viability of trophozoites after 48 h incubation with different concentrations of resveratrol was evaluated under light microscope by Trypan blue exclusion. IC_50_ was calculated as described in Materials and Methods. Values represent the mean ± standard error of three independent experiments.

Damage produced by resveratrol on *E*. *histolytica* trophozoites was time- and dose- dependent (Fig 1). Cell growth was arrested by 72 μM resveratrol after 12 h incubation, but this dose had little effect on cell viability, cellular shape and motility. |After 48 h incubation with 220 μM resveratrol, 50% of trophozoites appeared death, whereas more than 90% were killed by 440 and 880 μM, respectively (Fig 1). Thus, the IC_50_ was determined as 220 μM and it was used for the majority of the experiments presented here. Trophozoites incubated for the same periods with 0.4% ethanol, the vehicle used for the drug, exhibited 100% viability (Fig 1).

**Fig 2:**
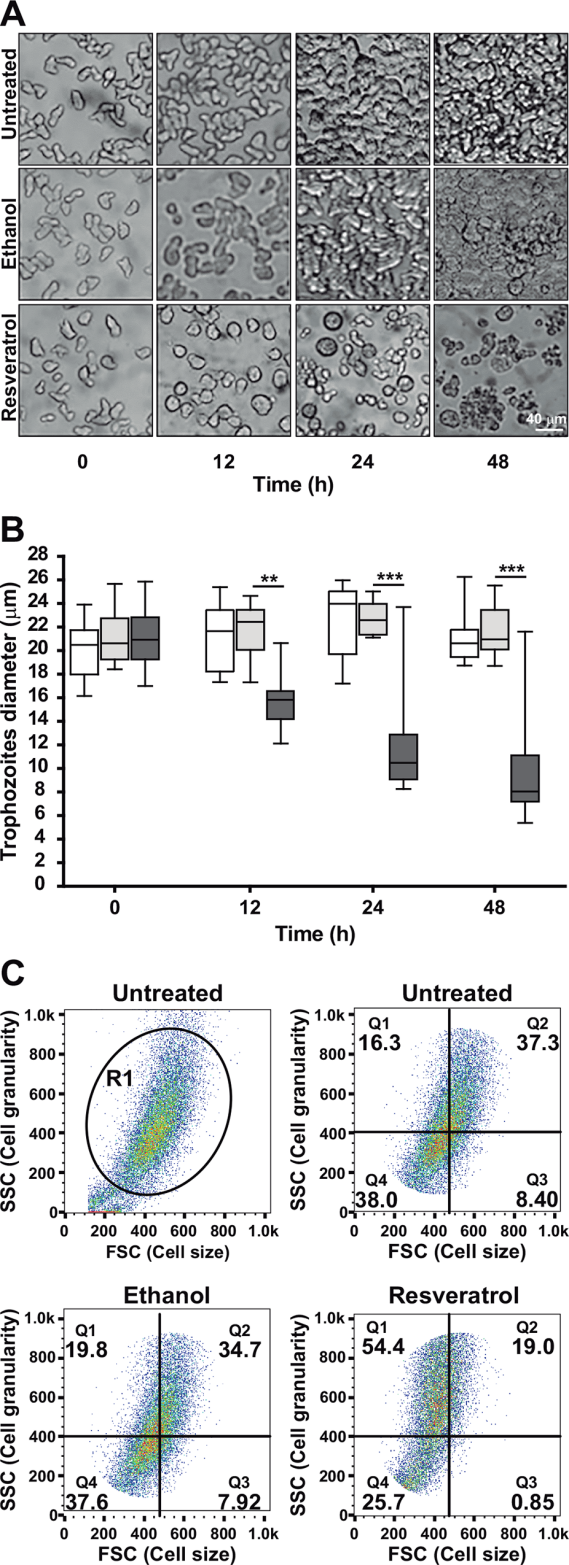
Effect of resveratrol in trophozoites morphology. **A)** Light microscopy images of untreated and IC_50_ resveratrol-treated trophozoites. **B)** Size of trophozoites during different incubation times measured in images obtained by light microscopy. Boxes represent 50% of population containing the median of three independent experiments. Bars indicate the maximum and minimum sizes of the other 50%. □ Untreated trophozoites. ■ 0.4% ethanol-treated trophozoites. ■ IC_50_ resveratrol-treated trophozoites. **p<0.01, ***p<0.001. **C)** Flow cytometry of size (forward scatter) and granularity (side scatter) of trophozoites incubated 48 h with IC_50_ resveratrol. R1 represents the gate of trophozoites population selected for these experiments. Untreated and 0.4% ethanol-treated trophozoites were used as controls.

Under light microscopy, untreated and ethanol-treated trophozoites appeared pleomorphic and with diameters between 16 and 26 μm (mean: 21 μm). After 12 h incubation with IC_50_ resveratrol, 50% of the cells presented diameters between 14 and 16 μm (Fig 2A and 2B). However, more than 98% of trophozoites remained alive and they excluded Trypan blue. At 24 and 48 h, resveratrol-treated trophozoites exhibited 75 and 50% viability, respectively, they were observed rounded, smaller (8 to 12 μm in diameter, respectively), detached from substrate, clustered and with poor motility (Figs 1A, 2A and 2B), whereas untreated and ethanol-treated cells reached confluence (Fig 2A).

The trophozoite shrinkage produced by 48 h incubation with resveratrol was confirmed by flow cytometry, measuring cell size by the decrease in forward scatter, and cell granularity by light scattered at 90° angle in side scatter. Resveratrol-treated trophozoites showed a reduction in cell size (Fig 2C), confirming the data obtained above (Fig 2B). Eighty percent of resveratrol-treated cells were located in quadrants (Q) 1 and 4, where the smaller cells were sorted (Fig 2C); whereas, 57.4% of ethanol-treated trophozoites were dispersed there (Fig 2C). In addition, 73.4% of resveratrol-treated trophozoites were found in Q1 and Q2; corresponding to cells with a higher granularity, whereas 54.5% of the ethanol-treated cells were located also in these Qs (Fig 2C). These results confirmed that resveratrol shrank trophozoites and produced granularity in their cytoplasm.

### Resveratrol-treated trophozoites present ultrastructural alterations

**Fig 3:**
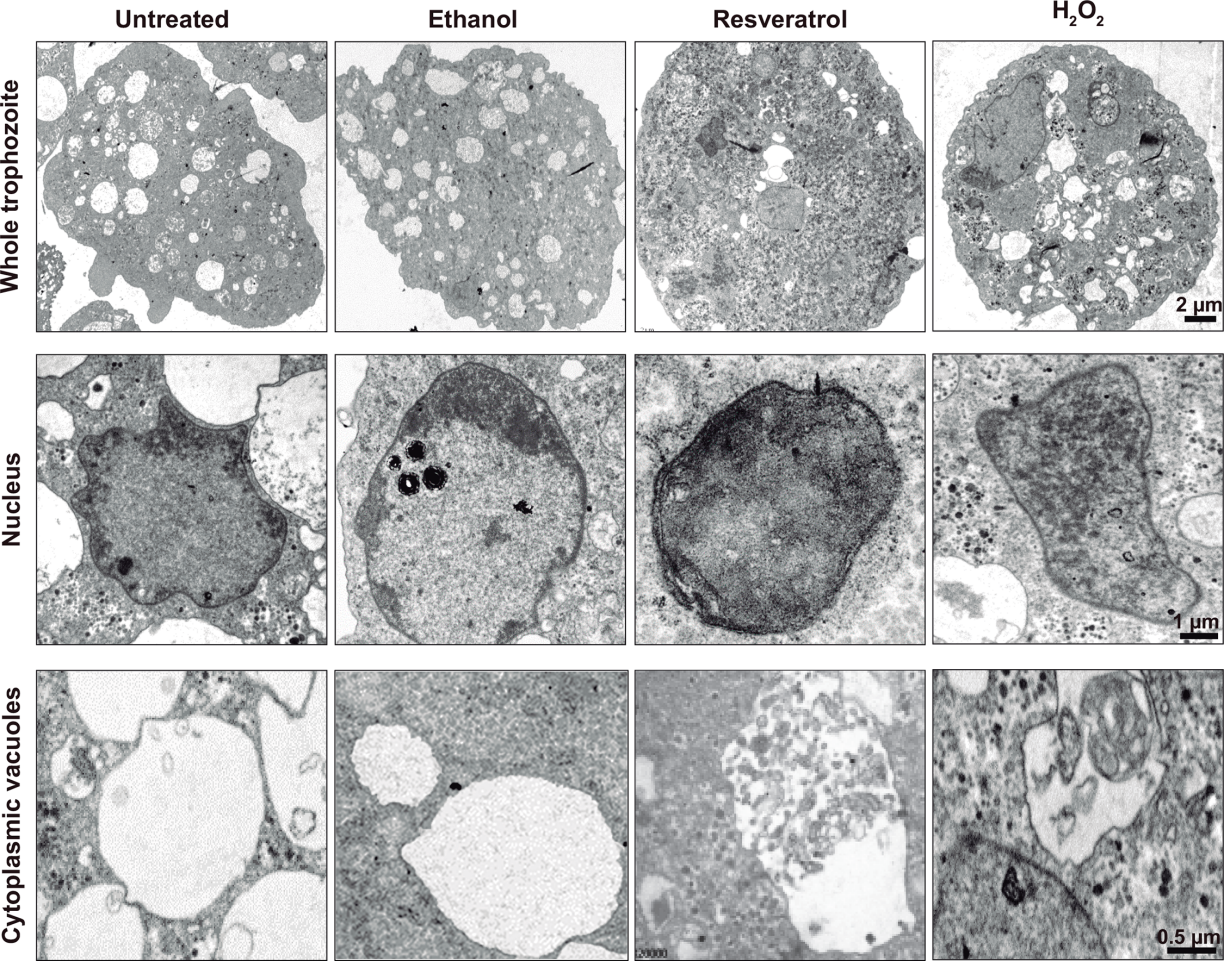
Ultrastructure of resveratrol-treated trophozoites. TEM of untreated, 0.4% ethanol-, IC_50_ resveratrol- and 0.5 mM H_2_O_2_-treated trophozoites.

The ultrastructural alterations provoked by resveratrol in trophozoites were studied by TEM. Resveratrol-treated trophozoites appeared rounded and with less cytoplasmic vacuoles, but with a large number of debris-containing vacuoles. They presented granularity and a higher electrodensity in the cytoplasm, their chromatin appeared diffuse and distributed in the whole nucleus. In comparison, untreated and ethanol-treated trophozoites exhibited normal ultrastructure with the chromatin condensed close to the inner nuclear membrane (Fig 3). Trophozoites incubated with H_2_O_2_, used as positive control, also exhibited ultrastructural alterations (Fig 3). The ultrastructural damage of trophozoites confirmed the results obtained by flow cytometry (Fig 2) and suggested that resveratrol-treated trophozoites were under stress. Besides, the presence of debris-containing vacuoles suggested autophagy, whereas the abnormally condensed chromatin could imply that resveratrol provokes apoptosis-like death.

### Resveratrol triggers oxidative stress in trophozoites

**Fig 4:**
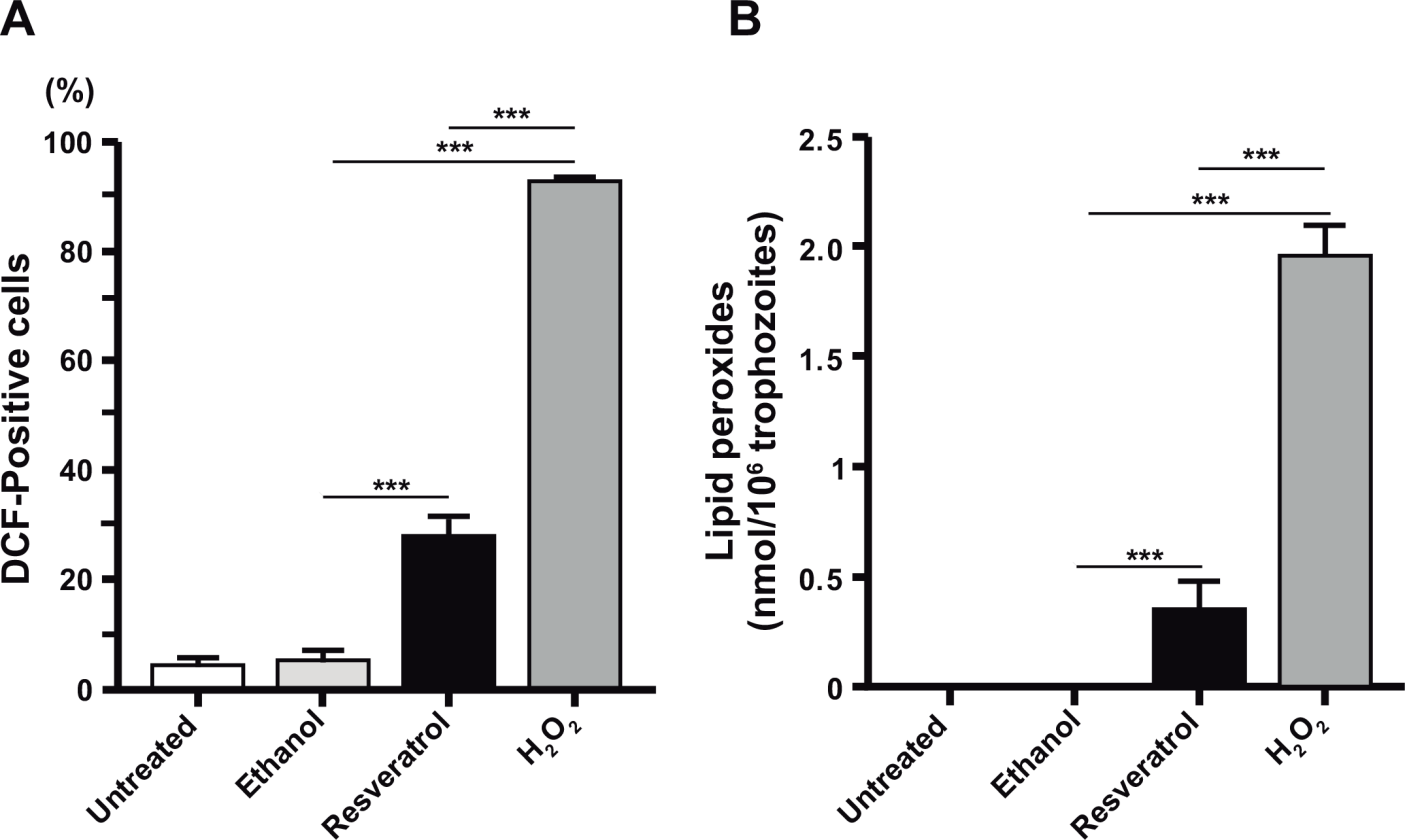
Oxidative stress produced by resveratrol in trophozoites. **A)** ROS production in trophozoites untreated or treated with IC_50_ resveratrol. Quantification was performed by flow cytometry using the DCFDA reagent. **B)** Lipid peroxides in total lipids of trophozoites. 0.4% ethanol- and 0.5 mM H_2_O_2_-treated trophozoites were used as controls. Values represent the mean ± standard error of three independent experiments. ***p<0.001.

Balance between ROS production and anti-oxidant enzymes mediates the fate of a cell. Depending on the extent of oxidative damage, cells will survive or will die by necrosis, autophagy or apoptosis. The high ROS production, such as superoxide (O_2_^•−^), hydroxyl radicals (HO^•^) and hydrogen peroxide (H_2_O_2_), causes injury to lipids, DNA and proteins [35]. To evaluate ROS production in resveratrol-treated trophozoites, we used the DCFDA reagent that after diffusion into the cell is deacetylated by esterases, generating DCF, which is oxidized by ROS [36]. These experiments showed that 28% of resveratrol-treated trophozoites were DCF-positive, whereas only 5% of ethanol-treated and untreated cells were DCF-positive; and 93% H_2_O_2_-treated trophozoites were positive (Fig 4A). Results suggested that resveratrol generated ROS production in trophozoites. However, we are aware that the only way to conclusively say that ROS kill amoebae is to use a ROS antagonist to show that it protects amoebae from resveratrol-induced damage.

ROS production affects, among other molecules, polyunsaturated fatty acids in cell membranes, inducing lipid peroxidation [37]. We investigated whether resveratrol affected trophozoites lipids. The spectrophotometry analysis of total lipids (corresponding to 1 x 10^6^ trophozoites), showed that drug-treatment produced 0.375 nmol of lipid peroxides; H_2_O_2_- treatment generated 1.8 nmol; and lipid peroxides were not detect in untreated and ethanol-treated trophozoites (Fig 4B). These experiments, together with the ROS production assays, strengthen the hypothesis that resveratrol provoked oxidative stress in trophozoites and damaged cell membranes lipids.

### Phosphatidylserine (PS) is externalized in *E*. *histolytica* trophozoites treated with resveratrol

**Fig 5:**
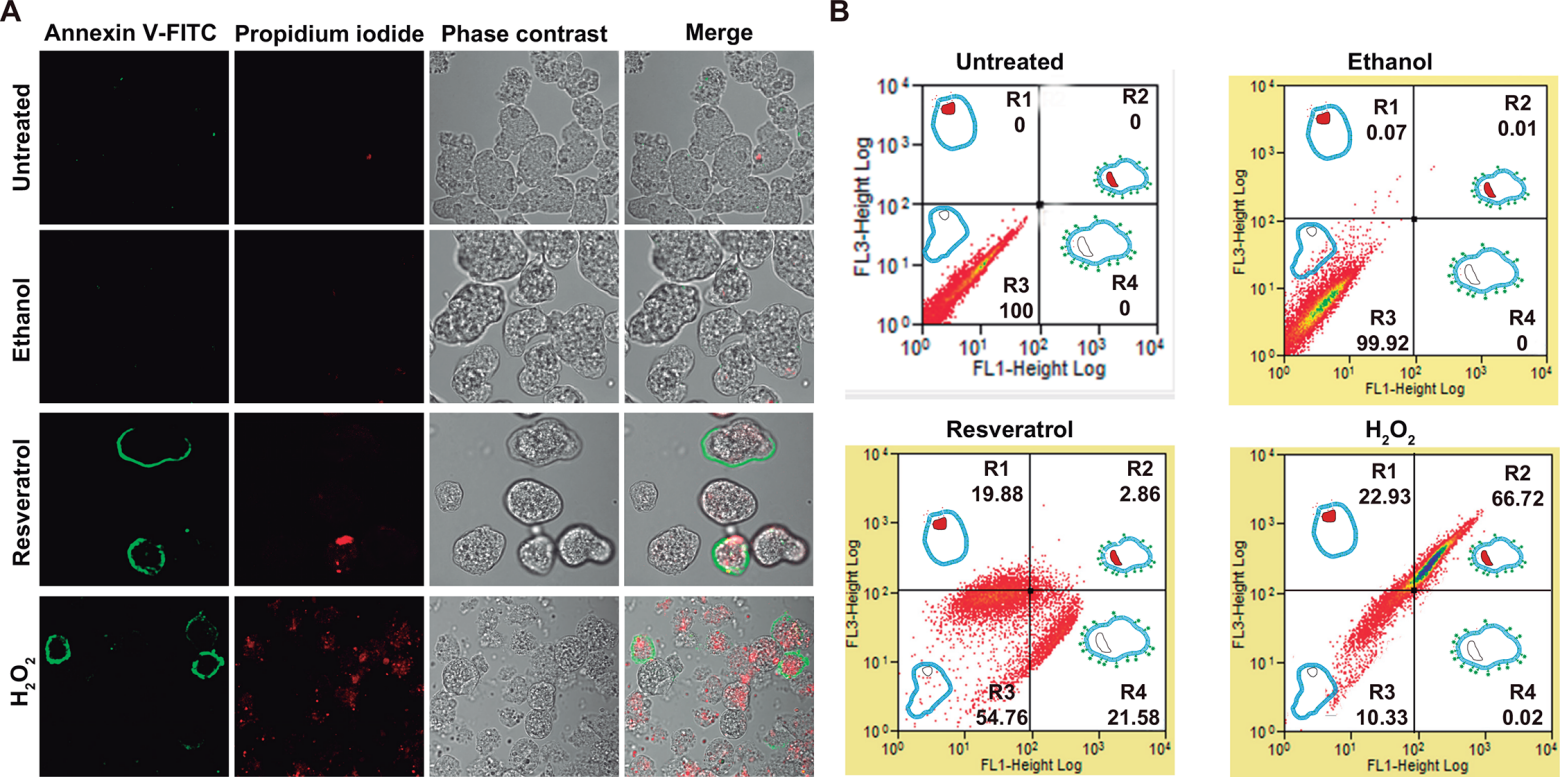
PS externalization produced by resveratrol in trophozoites. **A)** Confocal microscopy of untreated or IC_50_ resveratrol-treated trophozoites incubated with Annexin V-FITC and PI. Merge: fluorescence channels and phase contrast. **B)** Flow cytometry analysis of trophozoites incubated with Annexin V-FITC and PI. 0.4% ethanol- and 0.5 mM H_2_O_2_-treated trophozoites were used as controls. Q1: Trophozoite scheme with nucleus stained (red) representing entrance of PI. Q2: Trophozoite scheme representing PI stained (nucleus) and Annexin V plasma membrane stained. Q3: Trophozoite scheme representing plasma membrane integrity. Q4: Trophozoite scheme representing Annexin V (loss of plasma membrane asymmetry) stained without nucleus stained.

In eukaryotes, ROS production can conduct cells to apoptosis, characterized by cellular shrinkage, PS externalization, chromatin condensation, DNA fragmentation and caspases and calpain activation, among other events [37]. As PS externalization is used as a marker for apoptosis, we investigated whether PS was externalized in resveratrol-treated trophozoites, using Annexin V which has a high affinity for PS. Laser confocal images and flow cytometry experiments of non-permeabilized trophozoites revealed that Annexin V-FITC did not detect PS in untreated and ethanol-treated trophozoites. However, 21.58% of resveratrol-treated trophozoites exhibited PS exposed at the external plasma membrane, without showing plasma membrane rupture, as it was suggested by the trophozoites that were not stained by PI. However, 2.86% were stained by PI, suggesting loss of plasma membrane asymmetry (Fig 5A and 5B). These results could suggest that trophozoites that were not stained by PI, but exposed PS, are in an early apoptosis like event, whereas those that exhibited the nuclei stained by PI and exposed PS in the plasma membrane are in late apoptosis. By flow cytometry experiments, 66.72% of H_2_O_2_-treated trophozoites exposed PS (Fig 5B), but Annexin V was not detected in untreated and ethanol-treated trophozoites. These results suggested that resveratrol induced apoptosis-like death in *E*. *histolytica*.

### Resveratrol-treated trophozoites present DNA fragmentation

**Fig 6:**
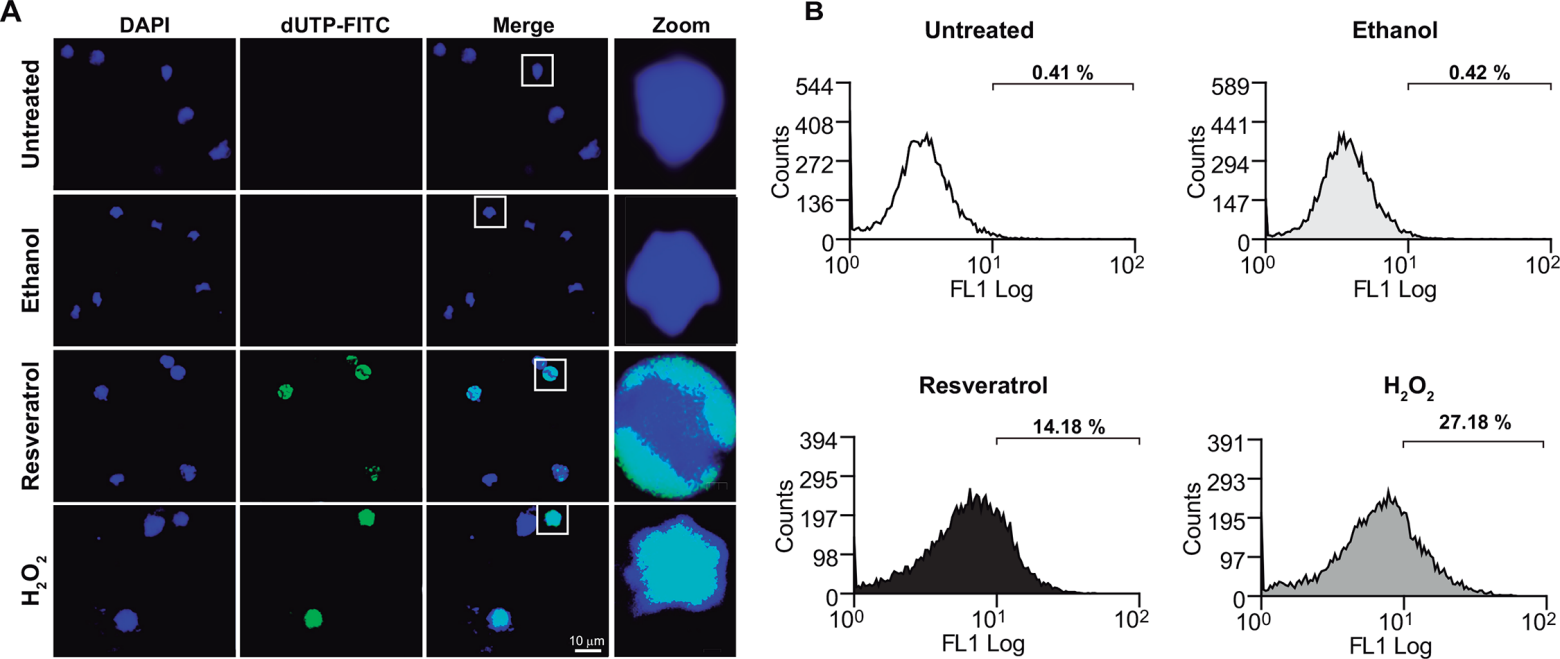
TUNEL assays in resveratrol-treated trophozoites. **A)** TUNEL assays of untreated or IC_50_ resveratrol-treated trophozoites using dUTP-FITC and visualized under the laser confocal microscope. Nuclei were stained with DAPI. Squares in merge images were magnified in the zoom panels. **B)** Flow cytometry analysis of dUTP-FITC-treated trophozoites. 0.4% ethanol- and 0.5 mM H_2_O_2_-treated trophozoites were used as controls.

To gain more evidence on resveratrol effect in trophozoites, we evaluated DNA integrity in cells treated with the drug. Confocal images showed that resveratrol- and H_2_O_2_-treated trophozoites exhibited a positive TUNEL signal (Fig 6A). In contrast, untreated and ethanol-treated cells showed a nuclear homogeneous DAPI staining, and no evidence for TUNEL was detected (Fig 6A). Flow cytometry assays showed that 14.18% of resveratrol-treated cells were positive for DNA fragmentation. Only 0.4% of untreated and ethanol-treated trophozoites presented DNA damage, while H_2_O_2_ provoked DNA rupture in 27.18% of trophozoites (Fig 6B). These results, together with the PS externalization assays, strongly suggested that resveratrol induces apoptosis-like death in *E*. *histolytica* trophozoites.

### Resveratrol increases cytosolic Ca^2+^ concentration and calpain activity, but diminishes SOD activity

**Fig 7:**
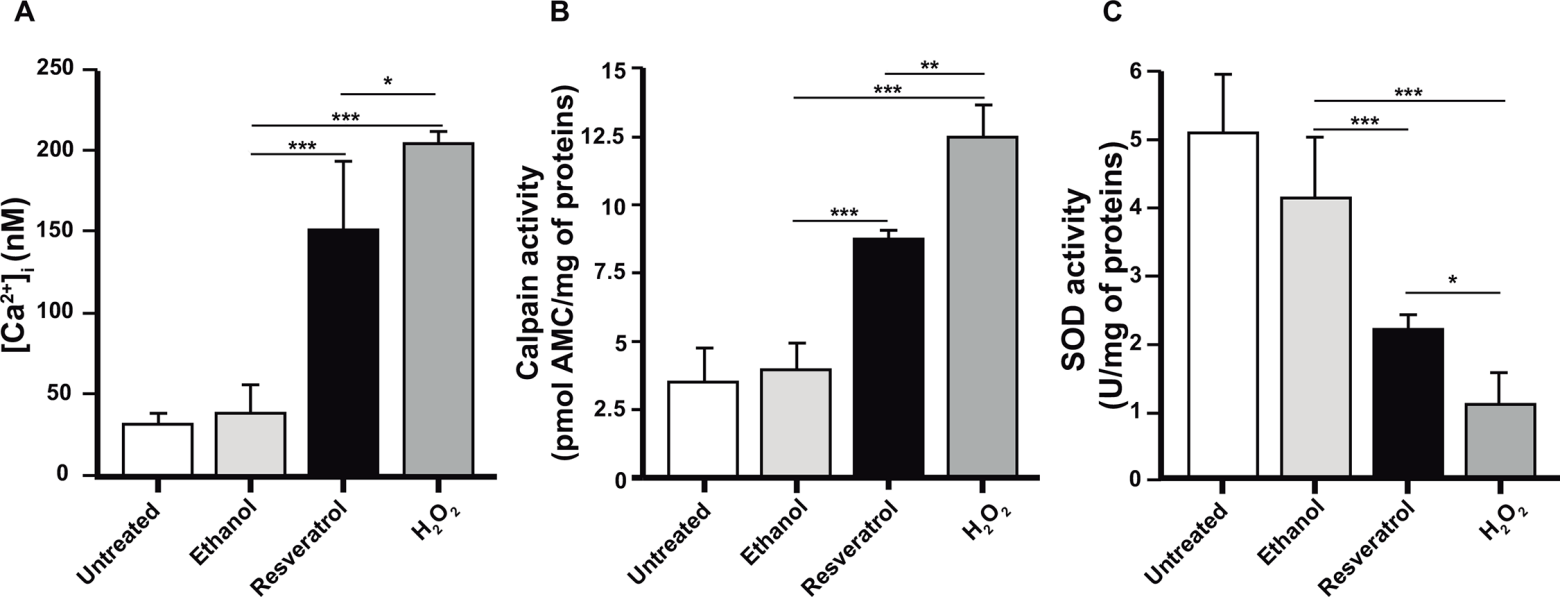
Biochemical changes in resveratrol-treated trophozoites. **A)** Cytosolic Ca^2+^ was measured in extracts of untreated or IC_50_ resveratrol-treated trophozoites, using Fluo-4 AM. **B)** Calpain activity of trophozoites extracts incubated with Suc-Leu-Leu-Val-Tyr-AMC was measured by fluorescence spectroscopy. **C)** SOD activity of trophozoite extracts was monitored by spectrophotometry using the SOD kit. 0.4% ethanol- and 0.5 mM H_2_O_2_-treated trophozoites were used as controls. Values represent the mean ± standard error of three independent experiments. ***p<0.001.

Oxidative stress and apoptosis produce biochemical changes that irreversibly damage proteins involved in calcium homeostasis. Thus, we used Fluo-4AM, a compound that binds Ca^2+^, to explore whether Ca^2+^ levels augmented in resveratrol-treated trophozoites. Fluorescence spectroscopy assays evidenced that cytosolic Ca^2+^ increased to 149 nM in resveratrol-treated trophozoites, in comparison with 31 and 39 nM showed by untreated and ethanol-treated cells, respectively. H_2_O_2_-treated trophozoites increased Ca^2+^ to 204 nM (Fig 7A).

In eukaryotes, caspases and calpain activation occurs when cytosolic Ca^2+^ concentration increases [37]. As *E*. *histolytica* has no canonical caspases, but has calpain-like enzymes [23,24], we investigated whether calpains were activated in resveratrol-treated trophozoites. Total extracts from trophozoites were incubated with Suc-Leu-Leu-Val-Tyr-AMC compound, which releases AMC as a result of calpain activity. These experiments evidenced that calpain released 8 pmol AMC/mg of total proteins in resveratrol-treated cells, whereas 3.7 pmol/mg were released in untreated trophozoites (Fig 7B).

SOD constitutes the first line of defense against ROS and converts O^-^_2_ in peroxide and molecular oxygen. We measured SOD activity in trophozoites extracts utilizing the WTS-1 reagent of the SOD assay. Resveratrol-treated trophozoites presented 2 U/mg of SOD, whereas untreated and ethanol-treated trophozoites presented 5 and 4 U/mg, respectively (Fig 7C). All our biochemical experiments confirmed that resveratrol induced apoptosis-like death in *E*. *histolytica* trophozoites.

### Resveratrol affects the *in vitro* virulence of *E*. *histolytica* and prevents encystment of *E*. *invadens*

**Fig 8:**
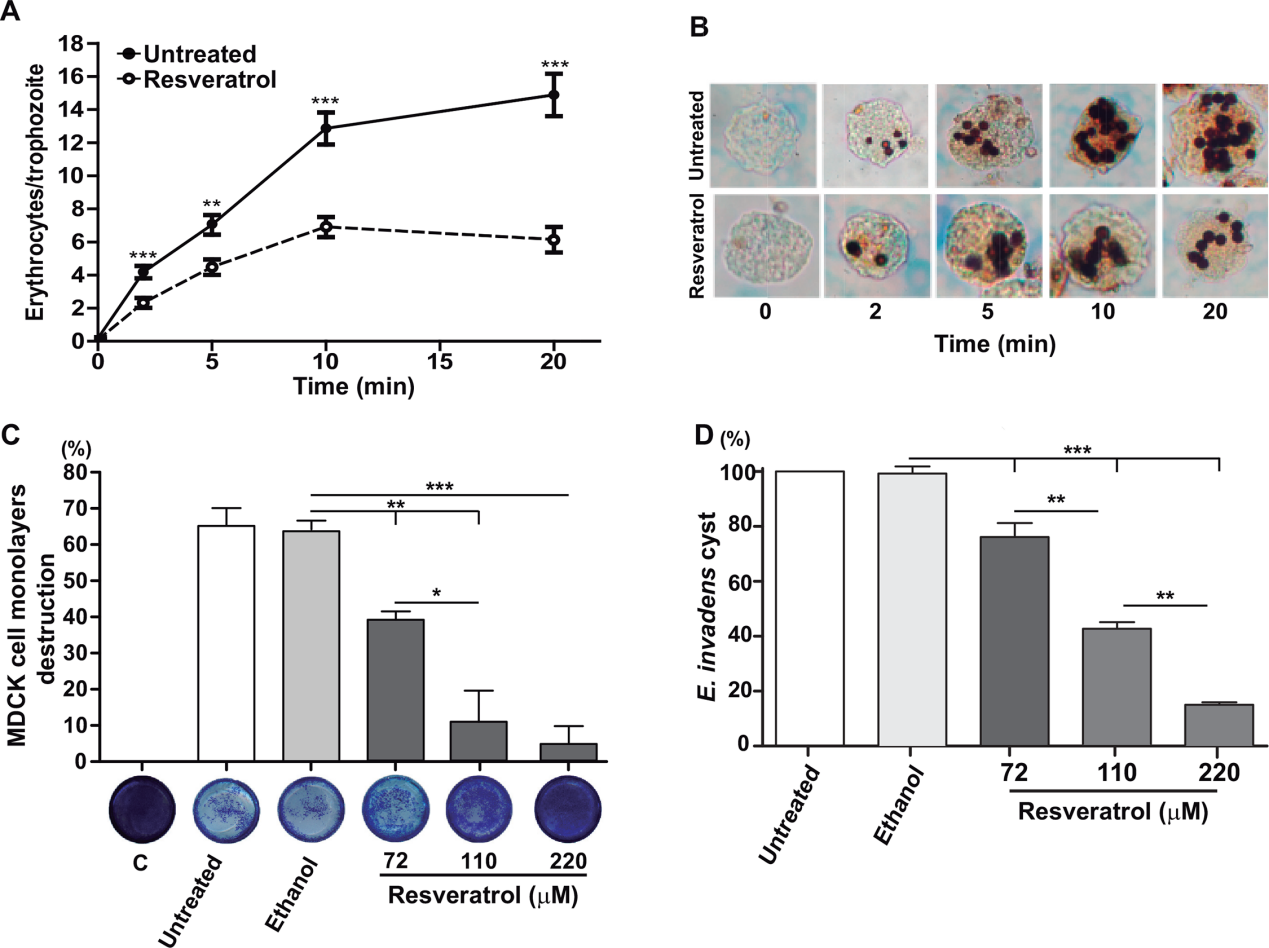
Effect of resveratrol in *in vitro* virulence of *E*. *histolytica* trophozoites and in *E*. *invadens* encystment. **A)** Ingested erythrocytes after different incubation times with untreated or resveratrol -treated (110 μM_,_ 12 h and washing out) trophozoites. Values represent the mean ± standard error of three independent experiments. **p<0.01, ***p<0.001. **B)** Representative images of trophozoites with ingested erythrocytes at different incubation times. **C)** Destruction of MDCK cell monolayers incubated with untreated or resveratrol-treated (110 μM_,_ 12 h and washing out) trophozoites. Bottom: representative images of MDCK monolayers incubated with trophozoites and stained with methylene blue. C: MDCK cells that were not in contact with trophozoites. 0.4% ethanol-treated trophozoites were used as positive controls. Values represent the mean ± standard error of three independent experiments. *p<0.05,** p<0.01,*** p<0.001. **D)** Cysts formed were counted under the epifluorescence microscope and number of cysts in untreated cells was taken as 100%. Values represent the mean ± standard error of three independent experiments., ** p<0.01,***p<0.001.

Trophozoites are professional phagocytes that ingest erythrocytes, epithelial cells and bacteria as nutrition sources. Additionally, phagocytosis is considered as a virulence factor in *E*. *histolytica* [38]. Thus, we evaluated the erythrophagocytosis rate of trophozoites that were incubated with 110 μM resveratrol for 12 h and whose viability was more than 95%. The number of ingested erythrocytes was counted in 100 trophozoites that were incubated with erythrocytes for different times. Results showed that the rate of phagocytosis diminished 50% in trophozoites pre-treated with 110 μM resveratrol for 12 h (Fig 8A), reaching a plateau 10 min after incubation with erythrocytes. Interestingly, the majority of trophozoites ingested erythrocytes, as another evidence of their viability (Fig 8B).

We also measured the ability of resveratrol-treated trophozoites to destroy MDCK cell monolayers. After incubation with MDCK cells for one hour, resveratrol-treated trophozoites (72, 110 and 220 μM for 12 h) destroyed 40, 12 and 5% of cell monolayers, respectively, whereas untreated and ethanol-treated trophozoites destroyed 65% (Fig 8C).

Cysts are the infective phase of *E*. *histolytica*, and if cysts are not produced, the disease can be controlled because transmission from individual to individual does not occur. However, it is not possible to consistently obtain *E*. *histolytica* cysts in the laboratory. Thus, to study the effect of resveratrol on encystment, we induced *E*. *invadens* trophozoites to encystment [39]. Number of cysts formed in encystment medium was counted after calcofluor staining. Percentage of cysts produced was calculated taken as 100% the cyst number produced in untreated and ethanol-treated cultures. Results showed that resveratrol inhibited cyst formation in a dose-response model (Fig 8D).

**Fig 9:**
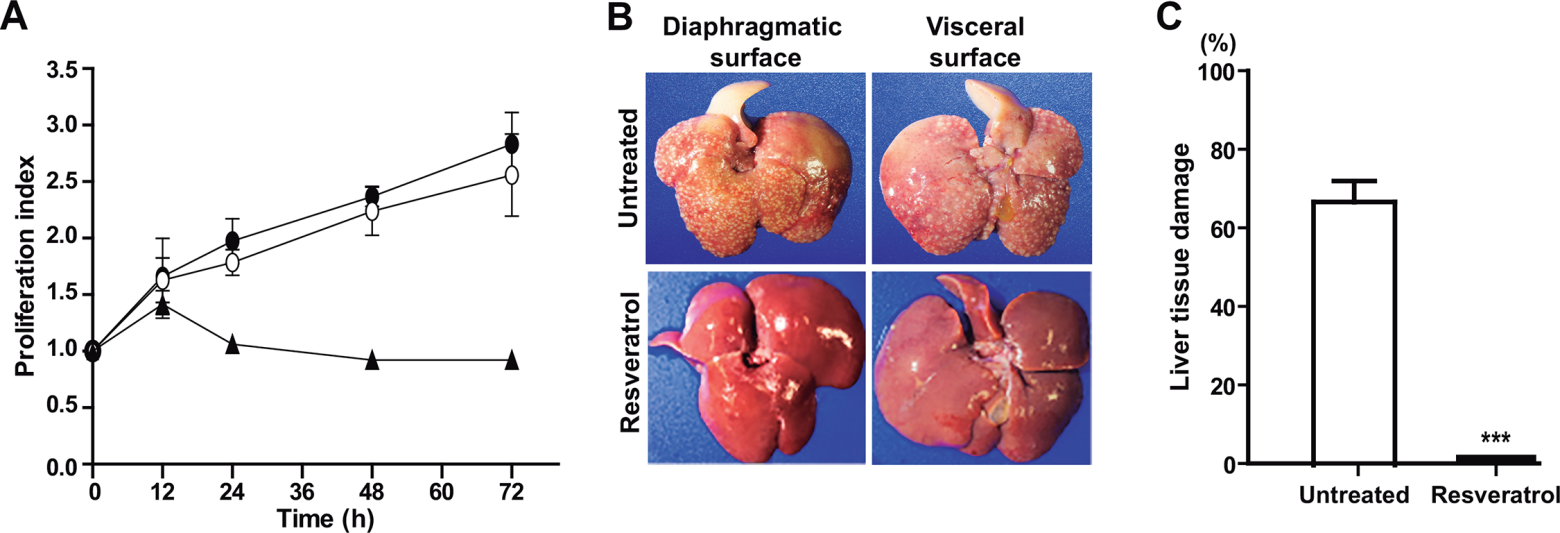
Hepatic abscess formation by resveratrol-treated and untreated trophozoites. **A)** Trophozoites were incubated with 110 μM resveratrol for 12 h, then they were changed to fresh TYI-S-33 medium without resveratrol and cell proliferation was measured at different times by spectrophotometry using the WST-1 reagent. -•- Untreated trophozoites. -○- Trophozoites pretreated with 110 μM resveratrol for 12 h and then incubated in fresh TYI-S-33 medium. -▲- Trophozoites cultured in the presence of 110 μM resveratrol during 72 h. **B)** Hamsters were intraportally inoculated with 3 x 10^6^ untreated or resveratrol-treated (110 μM, 12 h, drug removal) trophozoites. After seven days, livers were examined. **C)** Damage was evaluated as the weight of the abscesses formed divided by the weight of the whole liver, before the injured areas were removed. Values represent the mean ± standard error of liver damage in inoculated animals. n = 8. ***p<0.001.

Hamsters are one of the most used animal models to produce amoebic liver abscess and study the events related to this infection [33]. We investigated the effect of resveratrol on the *in vivo* virulence of *E*. *histolytica* trophozoites inoculated in hamsters. The viability of resveratrol-treated trophozoites (incubated for 12 h with 110 μM resveratrol, washed and then incubated with fresh medium) was analyzed by measuring cell proliferation using the WST-1 reagent, before the experiments were performed. Proliferation indexes of resveratrol-treated trophozoites and untreated trophozoites were similar, indicating that 12h of resveratrol-treatment did not affect cell viability. However, a cytostatic effect was observed in trophozoites that remained in the presence of 110 μM resveratrol during 72 h (Fig 9A). Interestingly, incubation for 12 h with resveratrol, followed by washing out of the drug, completely abolished the ability of trophozoites to produce hepatic abscesses in hamsters. Hamsters’ livers exhibited normal size and color (Fig 9B) and all internal organs presented normal appearance without any evident damage. In contrast, seven days after challenge, 100% of the animals inoculated with untreated trophozoites developed large abscesses (Fig 9B and 9C).

### Resveratrol presents amoebicidal activity in hamsters inoculated with virulent trophozoites

**Fig 10:**
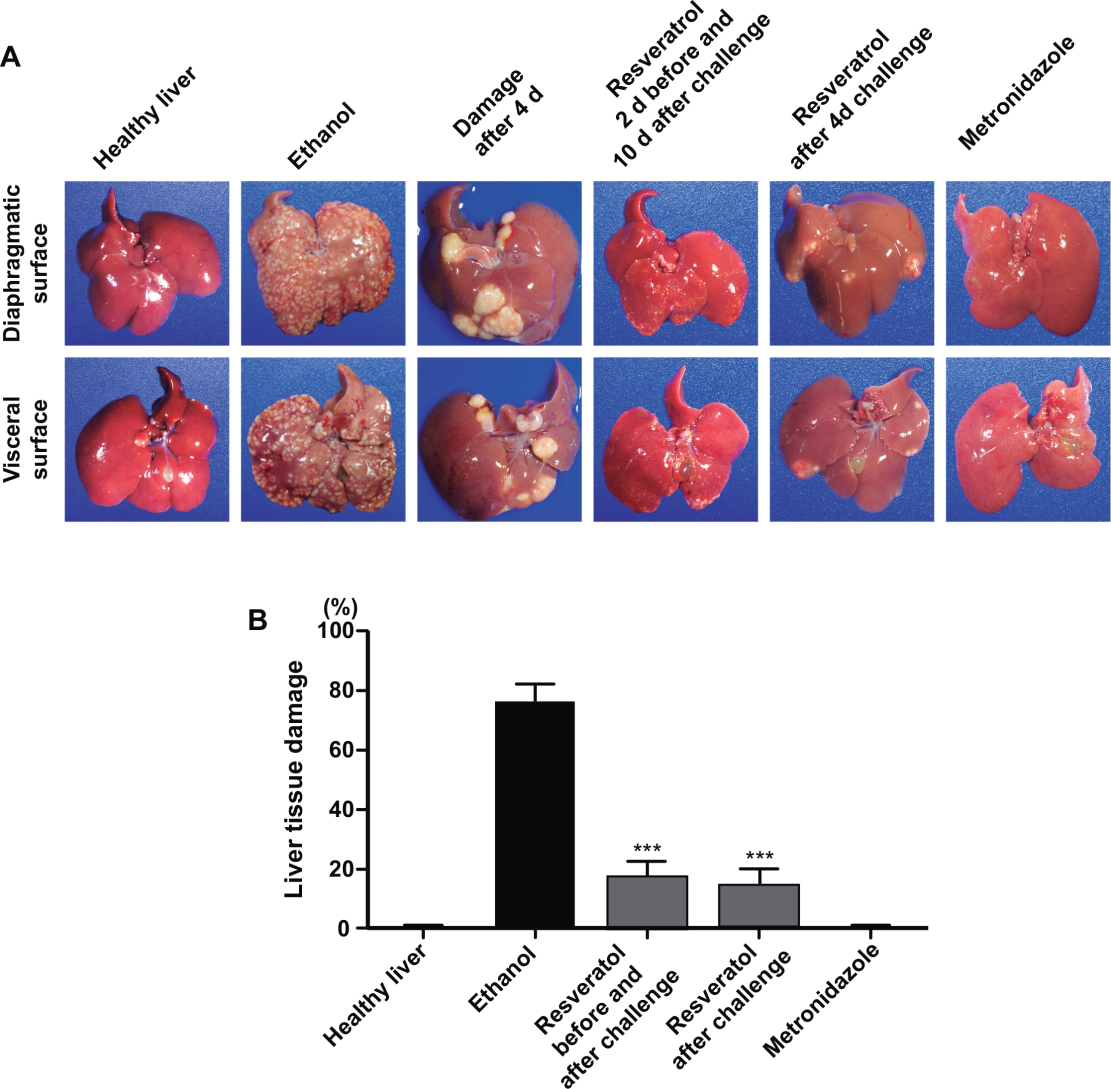
Effect of resveratrol administration in hamsters intraportally inoculated with virulent trophozoites. **A)** Healthy livers: Liver of animals not inoculated with trophozoites B) Ethanol: Livers of intraportally inoculated hamsters (3 x 10^6^ virulent trophozoites), treated with 50 μl of ethanol. Damage after 4 days: Livers of animals inoculated with virulent trophozoites and examined four days after challenge. Resveratrol 2d before and 10 d after challenge: Livers of animals treated with resveratrol (100 mg/Kg diluted in 50 μl of ethanol) each 8 h (2 days before and 10 days after inoculation) and examined ten days after challenge. Resveratrol after 4 d challenge: Liver of animals treated with resveratrol, as above, for ten days, starting four days after challenge when abscesses were already formed **B)** Damage was evaluated as the weight of the abscesses formed divided by the weight of the whole liver, before the injured areas were removed. As a negative control, animals were not inoculated with trophozoites (healthy liver). As a pharmacological control hamsters were treated with 20 mg/Kg of metronidazole. Values represent the mean ± standard error of liver damage in inoculated animals. n = 7. ***p<0.001.

Next, we evaluated the amoebicidal activity of resveratrol on animals (100 mg/Kg) inoculated with virulent trophozoites. Resveratrol (30 mg/Kg each 8 h, 2 days before and 10 days after inoculation) was orally administrated to hamsters intraportally inoculated with 3 x 10^6^ trophozoites. Ten days after challenge, animals that were not inoculated with trophozoites, but orally treated with pathogens-free water and ethanol presented healthy livers (Fig 10A). Ten days after challenge, animals inoculated with trophozoites and treated with pathogen-free water and ethanol developed huge abscesses that covered around 80% of the liver. In contrast, resveratrol-treated hamsters, two days before and ten days after challenge presented a mean of 17.28% liver damage (Fig 10A and 10B). Similar results were obtained in animals treated with resveratrol during ten days, but starting the treatment four days after challenging (after verifying the presence of liver abscesses in the positive control group). Abscesses were visible four days after challenge with virulent trophozoites in untreated animals. They appeared as huge isolated spots in 100% of animals used as positive controls for these experiments. Interestingly, a mean of 55% animals treated with resveratrol in both type of experiments did not present amoebic liver abscesses, 33% showed small abscesses located only in one lobule and covering 8% of the liver volume and 11% of the animals presented abscesses occupying 30% of the liver. All resveratrol-treated animals presented normal appearance and healthy spleen, gut, kidneys, lungs and other organs. As a pharmacological control we used for all experiments a group of animals that were treated with metronidazole to avoid the amoebic abscesses formation (Fig 10).

**Fig 11:**
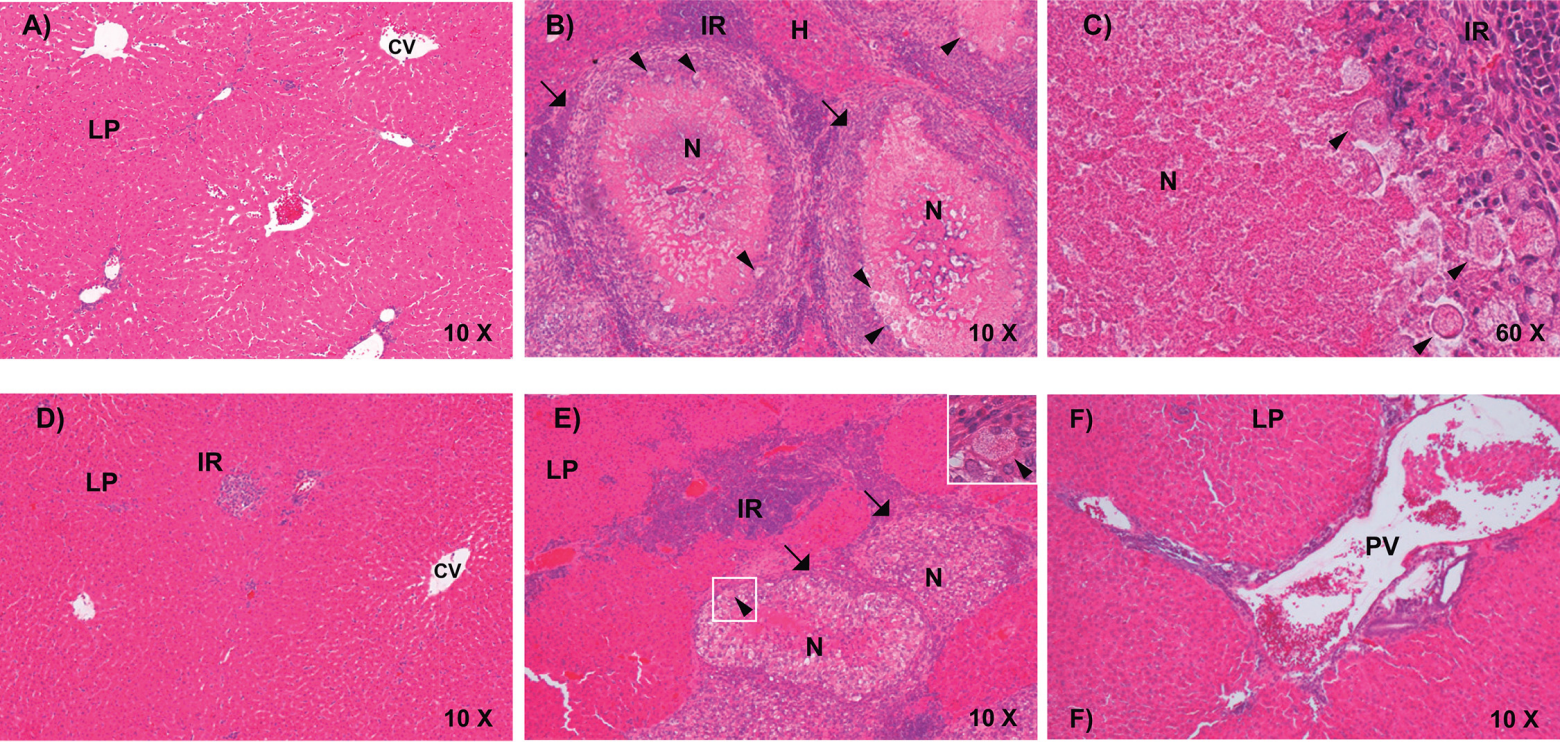
Histopathology of amoebic liver abscesses. 0.4 μm sections of hamster’s livers treated as described in Fig 10, were processed for hematoxylin-eosin staining and observed under light microscope. **A)** Animals not inoculated with trophozoites and orally treated with pathogens-free water. **B, C)** Hamsters intraportally inoculated with 3 x 10^6^ trophozoites and treated with 50 μl of ethanol each 8 h (2 days before and 10 days after inoculation). **B)** Granulomatous reaction representative of damage produced. **C)** Magnification of a granuloma. **D, E)** Hamsters intraportally inoculated with 3 x 10^6^ trophozoites and treated with 100 mg/Kg of resveratrol (diluted in 50 μL of ethanol) each 8 h (2 days before and 10 days after inoculation). **D)** Liver without damage after resveratrol-treatment of animals. **E)** Liver with small abscesses. Square: magnified area showing a trophozoite. Arrows: granulomas. **F)** Animals intraportally inoculated with 3 x 10^6^ trophozoites and treated with 20 mg/Kg of metronidazole each 8 h (2 days before and 10 days after inoculation). LP: liver parenchyma. CV: centrolobulillar vein. H: hepatocytes. N: necrosis area. IR: inflammatory reaction. PV: a branch of the portal vein. Arrowheads: trophozoites. Arrows: epithelioid cells.

To deeper analyze the liver damage, liver tissue sections were stained by hematoxylin-eosin and observed under the microscope. Animals without treatment that were not inoculated with trophozoites, but treated with pathogen-free water and ethanol, showed a normal liver parenchyma (Fig 11A). Ethanol-treated hamsters inoculated with virulent trophozoites presented important tissue damage with an intense granulomatous reaction. Liver necrosis increased by granulomas fusion and amoebae were observed in the border of them (Fig 11B and 11C). Interestingly, resveratrol-treated hamsters, using both protocols described here, that did not present hepatic abscesses, presented a healthy liver parenchyma (Fig 11D); whereas those animals that exhibited small liver abscesses showed an important decrease of granulomatous reaction, compared with the positive controls (Fig 11E). Inflammation area also diminished and few trophozoites were observed (Fig 11E). Metronidazole group showed healthy liver tissue after treatment (Fig 11F).

**Fig 12:**
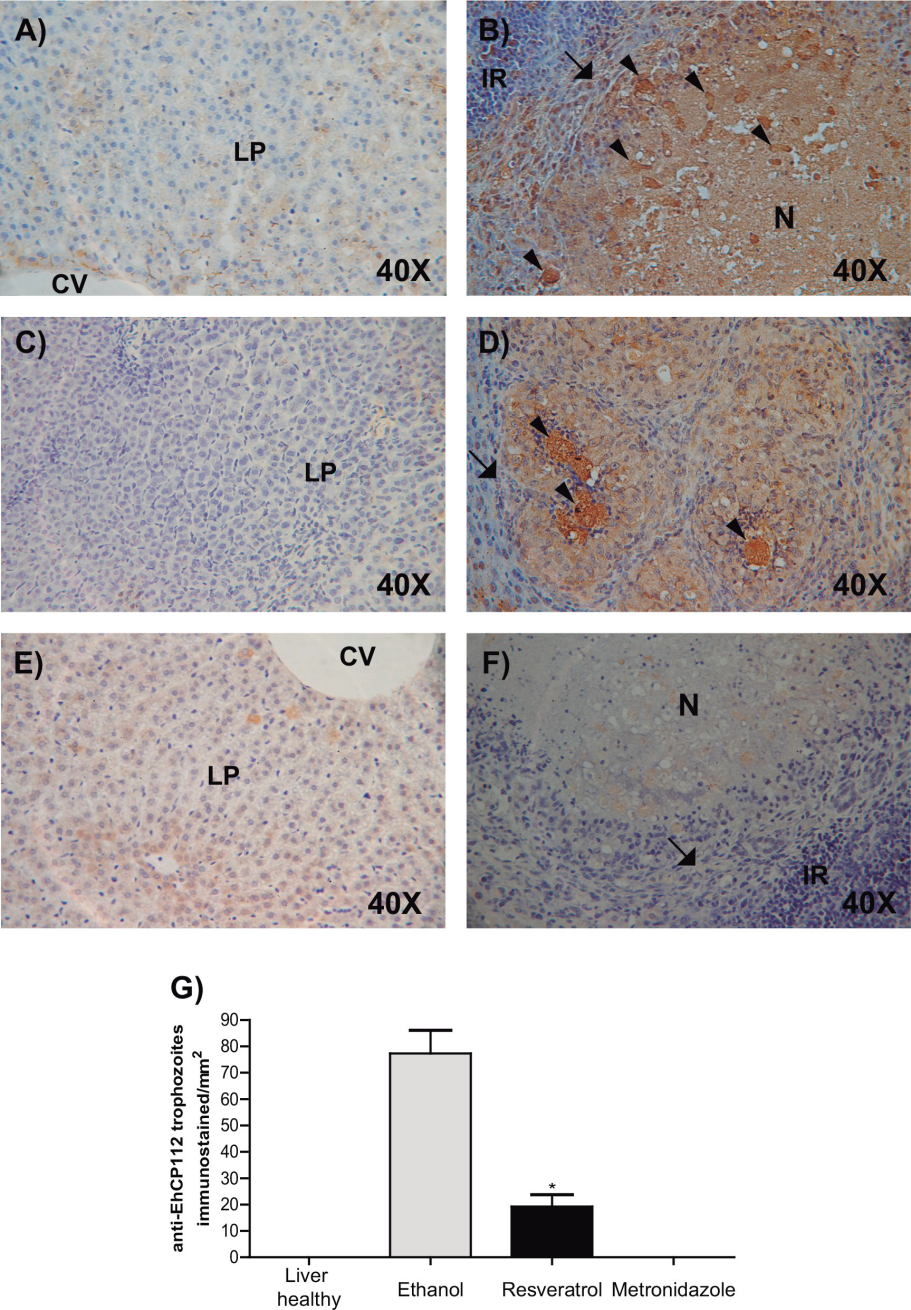
Immunochemistry of livers from hamsters inoculated with virulent trophozoites. Paraffin sections of livers from hamsters treated as in Fig 10. **A)** Non-challenged hamsters (healthy liver). **B)** Challenged hamsters treated with ethanol. **C)** Challenged hamsters treated with resveratrol that did no develop hepatic abscesses. **D)** Challenged hamsters treated with resveratrol that develop small abscesses. **E)** Challenged hamsters treated with metronidazole. **F)** Challenged hamsters treated with ethanol and developed only with the pre-immune serum. **G)** Parasitic burden quantified in 15 sections of livers from hamsters treated as above.

To evaluate the parasitic burden in the liver of untreated and resveratrol-treated animals (using both protocols described here), we carried out immunostaining assays, using an antibody against an EhCP112 polypeptide. EhCP112 is a cysteine protease forming part of the EhCPADH complex, involved in *E*. *histolytica* virulence [34]. To evaluate the parasitic burden, intact antibody-positive trophozoites were counted in 15 liver sections. The healthy livers from non-inoculated animals exhibited negative reaction (Fig 12A and 12G), similar to the negative control using only rabbit pre-immune serum (Fig 12F). Livers of animals treated with ethanol, presented a mean of 77.5 parasites/mm^2^ (Fig 12B and 12G). Hamsters treated with resveratrol with none or only small hepatic abscesses, exhibited a mean of 19.2 parasites/mm^2^ (Fig 12C, 12D and 12G), and metronidazole-treated animals gave negative reaction to the antibody (Fig 12G). In addition, in the granuloma area, a diffuse antibody-staining was observed, suggesting trophozoite rupture or EhCP112 secretion, as it has been reported [40].

## Discussion

Neglected parasitic diseases are extended around the world, mainly in Africa, Asia and Latin America poor countries [41]. Endoparasites, including protozoa and helminthes, affect more than 30% of the human population [41], and their eradication it is not in sight. Amoebiasis, caused by *E*. *histolytica*, is the third major human neglected parasitic diseases [42]. It is controlled mainly by metronidazole and its derivatives that are toxic for humans [43]. Natural products that have been used in poor countries for centuries to treat several diseases could be an alternative to treat amoebiasis and other parasitic infections. Between 1981 and 2006, 1,184 new drugs were registered, of which, 28% were natural products or their derivatives [44]. These data suggest that it is the time to look at biodiversity and ancestral knowledge for other strategies to control infectious diseases, including amoebiasis [9]. Here, we found that resveratrol, a nontoxic to humans natural compound, present in red wine, grapes, nuts and many medicinal plants [45], is cytotoxic for *E*. *histolytica* trophozoites in a dose- and time- dependent manner. As the innocuousness of resveratrol in mammals has been demonstrated using up to 2 g/Kg of the drug [13], resveratrol could be a good candidate against amoebiasis.

Besides, a number of favorable pharmacological activities in mammals, including anti-cancer properties, resveratrol kills several protozoa and helminthes, such as *Leishmania* (153.2 μM dose) [46], *Trichomonas vaginalis* (25 to 100 μM dose) [47],*Trichinella spiralis* (12.5 to 200 mg/ml dose) [48], *Acanthamoeba castellanii* (876 μM dose) [49] and *Balamuthia mandrillaris* (250 μM dose) [50], just to mention some. These data indicate that resveratrol dose vary depending on the organism [51]. Interestingly, several human eukaryotic parasites share some features, such as a high metabolic activity, uncontrolled cell division and development of resistance to the host defenses [52]. This can explain why, resveratrol can also be used against several parasites, bacteria and fungi [46,51,53]. In addition, *E*. *histolytica* trophozoites resemble to cancer cells in two facts: that they do not present contact growth inhibition and they can migrate in the body to colonize different tissues.

In some parasites, the effect of resveratrol is due to the interference with the respiratory chain in mitochondria or in mitochondria-like organelles. In *Philasterides dicentrarchi*, a scuticociliate pathogen of turbot, resveratrol causes oxidative stress, inhibition of antioxidant enzymes and morphological alterations in the mitochondria [17,54]. In *T*. *vaginalis* the action mechanism involves induction of hydrogenosome dysfunction [47]. In *Trypanosomatides*, the kinetoplast is a main target of the drug [55]. *E*. *histolytica* trophozoites do not possess canonical mitochondria, although cytoplasmic organelles with mitochondrial-like enzymes (Cpn60, Cpn10, Hsp70, MCF and PFO enzymes [56,57,58]) and others with DNA [59,60] have been reported. However, more studies are necessary to determine first the function of these structures and then, define whether resveratrol affects them.

Resveratrol provoked accumulation of ROS in *E*. *histolytica* trophozoites and possibly from this event, the apoptosis-like death observed in resveratrol-treated trophozoites was induced. ROS act as signaling molecules that trigger proliferation, autophagy and apoptosis [19]. However, the ROS production and its effects caused by resveratrol in trophozoites might be deeper investigated.

Autophagy is involved in intracellular degradation, energy recycling and stress events, such as periods of nutrient deprivation, when cell needs to obtain amino acids and other nutrients [61].

In autophagy, the concerted participation of Atg proteins is necessary for the autophagosomes membrane formation [61] and in some parasites, Atg8 and Atg12 conjugation systems are enough for autophagy [62]. Even when *E*. *histolytica* has the Atg system, we could not detect EhAtg8-PE conjugation or autophagosomes in resveratrol-treated trophozoites. Apoptosis has been studied in protozoa, including *Leishmania*, *Trypanosoma*, *Trichomonas* [63] and *E*. *histolytica* [64]. Even when *E histolytica* trophozoites do not have canonical caspases [24], here we demonstrated that apoptosis-like death occurs in resveratrol-treated trophozoites. Different experimental approaches, including PS externalization, DNA fragmentation, increase of cytosolic Ca^2+^, calpain activation and decrease of SOD activity, support this assumption.

Of the major interest was the effect of resveratrol on trophozoite virulence. The ability of trophozoites to destroy cultured cells and their rate of phagocytosis are two parameters to evaluate the *in vitro* virulence of *E*. *histolytica* strains. Here we showed that pre-incubation of trophozoites with 110 μM resveratrol produced a decrease in the rate of erythrophagocytosis and in cytopathic effect. Similar findings in relation to the effect of resveratrol on cytopathic effect of trophozoites on cell cultures were obtained for *A*. *castellanii* and *B*. *mandrillaris* [49,50]. Interestingly, pre-incubation with resveratrol of *E*. *invadens* trophozoites, inhibited encystment. Even when we could not prove this in *E*. *histolytica* trophozoites, resveratrol would be a good candidate to prevent cyst transmission.

*E*. *histolytica* trophozoites pre-treated with resveratrol were not able to produce liver abscesses in hamsters. Proliferation index experiments demonstrated that resveratrol-treated trophozoites (110 μM, 12 h) remained viable after drug removal, and therefore, when they were inoculated into the animals. Similarly, they were able to ingest red blood cells. These results also suggested that apoptosis-like death of trophozoites was triggered after 12 h incubation with resveratrol and before that, trophozoites remain viable, but unable to express virulence *in vitro*.

The amoebicidal effect of resveratrol was further demonstrated in hamsters inoculated with virulent trophozoites. Abscesses formation was dramatically diminished by oral administration of resveratrol in two distinct protocols: for two days before and ten days after and for ten days starting four days after intraportally inoculation of a high number of virulent trophozoites. The parasitic burden in the livers significantly decreased in resveratrol-treated animals, as it was shown by immunostaining using the anti-EhCP112 antibody. These results suggested that resveratrol could be proposed, after clinically testing, as an alternative to avoid amoebic hepatic abscesses. In addition, resveratrol derivatives must be tested as other options, as other authors have been done for cancer treatment and against other parasites [65]. Resveratrol and its derivatives could be a better choice than metronidazole to defeat amoebiasis, because resveratrol’s harmlessness in mammals has been demonstrated [13].

In conclusion, here, we introduced an old compound, the resveratrol, as a promising novel drug to defeat amoebiasis, because it affected the *in vitro* and *in vivo* virulence of the trophozoites and their capacity of encystment. The mechanisms through which resveratrol exerts its effect on *E*. *histolytica* involve the arrest of cell growth and the generation of oxidative stress which eventually triggered the apoptosis-like in trophozoites.
